# Association between characteristics of nursing teams and patients' aggressive behavior in closed psychiatric wards

**DOI:** 10.1111/ppc.13099

**Published:** 2022-05-03

**Authors:** Paul Doedens, Jentien Vermeulen, Gerben ter Riet, Lindy‐Lou Boyette, Corine Latour, Lieuwe de Haan

**Affiliations:** ^1^ Department of Psychiatry Amsterdam UMC, location Academic Medical Center Amsterdam The Netherlands; ^2^ Urban Vitality – Centre of Expertise, Faculty of Health Amsterdam University of Applied Sciences Amsterdam The Netherlands; ^3^ Department of Cardiology Amsterdam UMC, location Academic Medical Center Amsterdam The Netherlands; ^4^ Department of Clinical Psychology University of Amsterdam Amsterdam The Netherlands; ^5^ Arkin Amsterdam The Netherlands

**Keywords:** aggression, mental health services, nurses, personality, psychiatry

## Abstract

**Purpose:**

Estimate the effect of nursing, shift, and patient characteristics on patients' aggression.

**Design and Methods:**

Follow‐up study on a closed psychiatric ward was performed to estimate the effect of nursing team characteristics and patient characteristics on the incidence of aggression.

**Findings:**

The incidence of aggression (*n* = 802 in sample) was lower in teams with >75% male nurses. Teams scoring high on extraversion experienced more verbal aggression and teams scoring high on neuroticism experienced more physical aggression. Younger patients and/or involuntarily admitted patients were more frequently aggressive.

**Practice Implications:**

These findings could stimulate support for nurses to prevent aggression.

## 1 INTRODUCTION

Aggressive behavior on psychiatric wards imposes a high risk of adverse outcomes for patients and staff (Bhavsar & Bhugra, 2018; de Mooij et al., 2015; Marcus et al., 2018; Thibaut et al., 2019). Aggressive behavior varies in manifestation, ranging from verbal aggression (e.g., shouting and threatening) to physical assault (Renwick et al., 2016). Nurses in closed psychiatric wards are at high risk of encountering aggressive behavior; more than half of them are victims of assault by patients during their career (Jang et al., 2021; Odes et al., 2021; Spector et al., 2014). Aggressive behavior toward nurses on psychiatric wards causes stress, anxiety, and injuries (Hilton et al., 2021; Needham et al., 2005). Subsequently, aggressive behavior is the main reason for nurses to use coercive measures (e.g., seclusion or restraint) (Cowman et al., 2017; Laukkanen et al., 2019). Coercive measures are also associated with serious adverse events (Funayama & Takata, 2019; Kersting et al., 2019). If we gain more insight into factors causing aggressive behavior, we can use it to reduce or prevent aggressive behavior.

Several meta‐analyses investigated which patient characteristics influence the incidence of aggressive behavior, such as male sex, young age, and/or involuntary admission (Iozzino et al., 2015; Weltens et al., 2021). Although highly relevant, concentrating solely on patient characteristics to assess the risk of aggressive behavior is a one‐sided strategy. The role of nursing characteristics can provide further insight in the risk or aggressive behavior (Ayhan et al., 2021; Lamanna et al., 2016; Weltens et al., 2021). Salzmann‐Erikson and Yifter (2019) found that nurses with longer employment encountered less aggressive patient behavior during their shift. They also reported that most aggressive incidents occurred in the evening shift (Salzmann‐Erikson & Yifter, 2019). Schlup et al. (2021) reported a lower self‐reported incidence of aggression with more experienced nurses. Başoğul et al. (2019) found that nurses with stronger needs for positive interaction with others reported more verbal aggression (Başoğul et al., 2019). While most authors reported results of verbal and physical aggression together, others analyzed verbal and physical aggression separately and found (small) differences in risk factors. Bowers, Allan et al. (2009) reported an association between the presence of student nurses and verbally aggressive patient behavior, but not with physical aggression. Başoğul et al. (2019) found that awareness of their own emotions by nurses was associated with less physical aggressive patient behavior. Besides factors of individual nurses, the literature describes several organizational factors that influence aggressive behavior of patients in mental health care, such as organizational justice, collaboration between staff, ward atmosphere, work functioning, and leadership (Bowers, 2009; Giménez Lozano et al., 2021; Magnavita et al., 2020; Pekurinen et al., 2017).

In summary, previous studies found several patient and nurse characteristics being associated with aggressive behavior of patients. However, most studies measured the association between individual nurse characteristics and their self‐reported experience with aggressive behavior. We propose to take into account nursing team, shift, and patient characteristics to estimate their effect on more reliably measured incidents of aggressive behavior. In the current study, we addressed the following questions:

(1) Which nursing team (e.g., personality traits, sex, and education), shift (e.g., patient‐staff ratio), and patient characteristics (e.g., sex and diagnosis) are associated with the incidence of aggressive patient behavior in closed psychiatric wards?
(2) Do associations differ between verbal aggression and physical aggression?

## 2 METHODS

### 2.1 Design

We performed a prospective 2‐year follow‐up study on a closed psychiatric ward.

### 2.2 Participants and setting

Our study was performed at the closed psychiatric admission ward of Amsterdam University Medical Centers (location Academic Medical Center) in the Netherlands. The ward is responsible for the involuntary admissions from densely populated, multicultural neighborhoods in Amsterdam. The ward had 12 patient rooms and two seclusion rooms, which serves as the last‐resort coercive measure in case of dangerous situations due to aggressive behavior. We included all patients admitted to the ward between January 1, 2013 and December 31, 2014. The majority of admissions were involuntary and related to acute psychiatric crises leading to danger, according to the local Mental Health Act. Nurses worked in three shifts with four registered nurses on 12 patients between 7:30 a.m. and 11:00 p.m. (day shift and evening shift) and two nurses at night. Student nurses work on a supernumerary basis.

### 2.3 Variables and measurements

We gathered nurses' baseline data with a case record form. Data collection on nurses consisted of sex, age, body mass index (BMI), physical stature, registration as a nurse (RN), highest education, full‐time or part‐time employment, duration of employment, and years of experience in mental health care. We defined physical stature as a nurse's subjective physical appearance, estimated on a 5‐point scale (very small, small, average, large, and very large). Three assessors independently rated stature; the observer agreement was moderate, Fleiss *κ* = 0.43.

Psychological measurements consisted of the Big Five personality traits (neuroticism, extraversion, openness, agreeableness, and conscientiousness) and a general feeling of safety during their work. We assessed personality traits using an online self‐report 60‐item Neuroticism Extraversion Openness Five‐Factor Inventory 3 (NEO‐FFI‐3) (McCrae et al., 2005). This instrument has adequate to good psychometric properties in patient groups and the general population (Hoekstra & De Fruyt, 2014; McCrae et al., 2005). Despite extensive literature research, we were unable to obtain a validated questionnaire to measure nurses' feelings of safety in psychiatric wards. Therefore, we used four questions with a 5‐point Likert scale about whether nurses generally felt safe in their organization, on their ward, with their colleagues, and with their manager.

We gathered baseline data on patients within a week after the start of their first admission to the ward during the study period, using the electronic health records. Patients' baseline data consisted of sex, age, length of admission, involuntariness of admission, primary and secondary diagnosis, whether the admission occurred after an aggressive incident, and current psychiatric status (based on the Health of Nation Outcome Scale [Wing et al., 1998] and Global Assessment of Functioning [Jones et al., 1995]).

We collected shift data in all shifts during the data collection period, that is, three times a day (day afternoon and night shift). To prevent bias due to underreporting of aggressive incidents, we screened daily nursing reports. The first author read all nursing reports for the admitted patients during the study period to find possible aggressive incidents. We performed outcome measurements for every patient during the entire study period using the Staff Observation Aggression Scale–Revised (SOAS‐R) (Nijman et al., 1999). Variables and measurements are described in detail in Supporting Information File 1.

### 2.4 Ethical considerations

Patients on closed psychiatric wards are a vulnerable population and researchers should be meticulous in protecting their rights (Helmchen, 2010). We requested the Medical Ethics Review Committee of our institution for approval according to the Medical Research Involving Human Subject Act (WMO). The committee concluded that formal approval of current study was not obligatory, as our study observed routine patient care and did not subject patients to additional procedures, behavioral rules or diagnostic testing (reference number A1‐12 17 0320). Because of the absence of impact on patients and the importance of our study aims, we were allowed not to seek active consent to re‐use patients' data for this study, according to the exception grounds of article 24 of the GDPR Implementation Act. To protect patients' privacy, only members of clinical staff performed data collection from the electronic health record. The current study used anonymized data in all analyses. Staff members were asked to participate on a voluntary basis and gave permission to use of their data in the analysis. Staff members were free to refuse participation and researchers did not communicate the (non)participation.

### 2.5 Statistical analysis

In this study, 98 different nurses, over the 2 years of follow‐up, formed 1299 different team compositions during 2190 shifts (three shifts during 730 days). Patients encountered many teams and many different nurses during their admission(s). Statistical literature refers to this nonhierarchical structure as cross‐classified data (Fielding & Goldstein, 2006). Cross‐classification signifies that our data do not have a simple hierarchical structure in which shift teams have fixed compositions of nurses and each patient receives care from a single nurse during the entire admission.

We analyzed the data by constructing a cross‐classified multilevel logistic regression model with occurrences of aggressive behavior as the dependent variable and nursing team, shift, and patient characteristics as independent variables. Team variables consisted of the mean score of the nurses present in a particular team, such as sex (two males and two females would yield 0.5), education, and personality traits. To improve the stability of the model, we categorized numerical variables using four categories for demographic variables and three categories (cutoffs of the 17th and 83rd centile values) for psychological categories, using the lowest category as a reference category. In STATA SE, version 15, we ran the runmlwin command to use MLwiN, version 3.02. We obtained starting values for the Markov Chain Monte Carlo analyses using penalized quasi‐likelihood estimates (PQL2). The burn‐in value was 2000 and the number of chains run was 20,000. We report odds ratios and their corresponding 95% credible intervals (95% CrI). We started our model with a high number (which are explained in Supporting Information File 1) of variables and retained variables if their p‐value was smaller than .20. We describe the STATA‐code in Supporting Information File 2.

## 3 RESULTS

### 3.1 Participants

For a summary of baseline characteristics of the nursing staff, we would like to refer to Table 1. In total, 98 nurses worked at least one shift during the study period. The majority were females (*n* = 60) and the mean age was 36 years (range 18–61). Incomplete case record forms (*n* = 7) were the main cause of missing data.

**Table 1:** Baseline characteristics of nurses (*n* = 98)

Characteristic		Missing data, *n*
Male	38 (38.8)	0
Age (years), mean (*SD*)	36.3 (13.5)	6
BMI, mean (*SD*)	23.4 (3.0)	7
Stature		19
Very small	2 (2.5)	
Small	14 (17.7)	
Average	44 (55.7)	
Large	18 (22.8)	
Very large	1 (1.3)	
Registered nurse	76 (77.6)	0
Bachelor of nursing	52 (54.7)	3
Years of employment, median (IQR)	2.0 (0–5.3)	3
Years of experience in psychiatry, median (IQR)	4.0 (0–17)	4
Employment		0
Permanent staff	26 (26.6)	
Student nurses	17 (17.3)	
Temporary staff (e.g. agency staff)	55 (56.1)	
Full‐time staff	59 (60.2)	0

*Note*: All numbers are *n* (%) unless indicated otherwise.

Abbreviations: BMI, body mass index; IQR, interquartile range; *SD*, standard deviation.

Table 2 contains the psychological trait scores of the nurses. Internal consistency was acceptable for neuroticism, extraversion, conscientiousness, and the general feeling of safety, however, low for openness and agreeableness. This is in line with findings in several samples in the population (Hoekstra & De Fruyt, 2014). Average team scores of the nurses were higher on extraversion and openness and lower on neuroticism, compared to reference categories in the general population (Hoekstra & De Fruyt, 2014). Thirty‐six nurses did not respond or refused participation in the psychological questionnaire. Most non‐responders (*n* = 33; 92%) were temporary staff members, who worked during fewer shifts than regular staff.

**Table 2:** Psychological traits of nursing staff (*n* = 62)

	Sample, mean (*SD*)	Cronbach's alpha	Reference group, mean (*SD*)
NEO‐FFI‐3			
Neuroticism	29.5 (6.1)	0.782	34.0 (7.5)
Extraversion	43.3 (6.1)	0.812	39.3 (5.8)
Openness	42.5 (5.2)	0.688	38.9 (5.7)
Agreeableness	45.2 (4.6)	0.617	41.1 (5.6)
Conscientiousness	44.7 (5.3)	0.765	43.4 (5.7)
General feeling of safety	15.4 (2.4)	0.899	

*Note*: Reference group based on a representative sample (n = 1715) from the general population (Hoekstra & De Fruyt, 2014).

Abbreviations: NEO‐FFI‐3, Neuroticism Extraversion Openness Five Factor Inventory 3d version; *SD*, standard deviation.

Table 3 contains a summary of patients' baseline characteristics. There were 224 patients, of whom 57 had multiple admissions. The majority of patients were male (*n* = 133; 59%) and their mean age at first admission was 39 years (range 18–80). Almost half of the patients (*n* = 108; 48%) showed aggressive behavior on the ward at least once.

**Table 3:** Baseline characteristics of patients at first admission (*n* = 224)

Characteristic	Aggression group (*n* = 108)	Nonaggression group (*n* = 116)	Group difference (*p* Value)
Male	67 (62.0)	66 (56.9)	0.496^a^
Age (years), mean *(SD*)	37.0 (13.7)	40.6 (13.2)	0.050^b^
Length of admission (days), median (IQR)	21 (0–42)	7 (5–21)	<0.001^c^
Involuntary admission	85 (78.7)	65 (56.0)	<0.001^a^
Primary diagnosis			0.003^d^
Psychotic disorder	81 (75.0)	70 (60.3)	
Bipolar disorder	16 (14.8)	13 (11.2)	
Other disorder	11 (10.2)	33 (28.5)	
Secondary diagnosis			
Substance abuse	53 (49.1)	39 (33.6)	0.021^a^
Personality disorder	10 (9.3)	15 (12.9)	0.405^a^
Intellectual impairment	8 (7.4)	7 (6.0)	0.791^a^
Admission after aggressive behavior	41 (40.0)	29 (25.0)	0.044^a^
First admission in mental health care	27 (25.0)	36 (31.0)	0.373^a^

*Note*: All numbers are n (%) unless indicated otherwise.

Abbreviations: IQR, interquartile range; SD, standard deviation.

^a^ Fisher's exact test, two‐sided.

^b^ Student's *t* test, independent samples.

^c^ Mann–Whitney *U* test, independent samples.

^d^ Chi‐square test, two‐sided (*df* = 2).

### 3.2 Outcomes

Tables 4a and 4b contain the observations of aggressive behavior. We documented 802 aggressive incidents during the data collection period. We divided aggressive incidents into verbal aggression (i.e., “verbal aggression” and “physically threatening” in the SOAS‐R) and physical aggression (i.e., “physical violence towards goods,” physical violence towards nursing staff,” and “physical violence towards fellow patients” in the SOAS‐R). We documented 438 incidents of verbal aggression only and 364 incidents of physical aggression.

**Table 4a:** Characteristics of aggressive behavior, measured by SOAS‐R

Provocation	*N* (%)	Means used by the patient	*N* (%)	Target of aggression	*N* (%)
No understandable provocation	227 (28.3)	Verbal aggression	438 (54.6)	Nothing/nobody	111 (13.8)
Provoked by other patient(s)	65 (8.1)	Ordinary objects (e.g., furniture)	126 (15.7)	Object(s)	113 (14.1)
Help with ADL	53 (6.6)	Parts of body (e.g., punching)	221 (27.6)	Other patient(s)	76 (9.5)
Patient being denied something	299 (37.3)	Dangerous objects (e.g., knife)	17 (2.1)	Patient self	14 (1.7)
Administration of medication	78 (9.7)			Staff member(s)	462 (57.6)
Other provocation	80 (10)			Other person(s)	26 (3.2)

Abbreviation: SOAS‐R, Staff Observation Aggression Scale‐Revised.

**Table 4b:** Consequences of aggressive behavior, measured by SOAS‐R

Consequences for victim(s)	*N* (%)	Measures to stop aggression	*N* (%)
None	148 (18.5)	None	53 (6.6)
Damaged objects	31 (3.9)	Talk to patient	353 (44.0)
Persons, felt threatened	569 (70.9)	Calmly brought away	24 (3.0)
Persons, pain	41 (5.1)	Enteral medication	69 (8.6)
Persons, injuries	13 (1.6)	Parenteral medication	25 (3.1)
		Physical restraint	8 (1.0)
		Mechanical restraint/seclusion	179 (22.3)
		Other	91 (11.3)

Abbreviation: SOAS‐R, Staff Observation Aggression Scale‐Revised.

### 3.3 Main results

In multilevel modeling, we observed high collinearity between nurses' experience in mental health care and nurses' age. We dropped age from the final analysis since we deemed experience a more important concept than age for our purpose. We dropped the following nursing team and shift characteristics from the final model due to their limited influence on the final model (since their odds ratios had *p* values ≥ .20): BMI, work experience, full‐time or part‐time employment, years of employment in the current hospital, patient‐staff ratio. Similarly, we dropped the following patient‐related characteristics: seclusion in patient's history, citizenship, current admission after aggressive behavior, first admission in mental health care, and admission during weekends. We present the results of the final regression model in Table 5.

**Table 5:** Results of the cross‐classified multilevel logistic regression analysis

	Aggressive behavior (*n* = 802)		Verbal aggression (*n* = 438)		Physical aggression (*n* = 364)	
	OR (95% CrI)	*p*	OR (95% CrI)	*p*	OR (95% CrI)	*p*
Nursing team characteristics						
Sex		<0.001		0.008		0.006
Only female nurses	Reference		Reference		Reference	
Mixed team, majority females (>50%)	0.701 (0.582–0.849)	0.002	0.670 (0.508–0.869)	0.004	0.754 (0.557–0.981)	0.040
Mixed team, majority males (50‐75%)	0.710 (0.563–0.884)	0.004	0.735 (0.542–0.974)	0.036	0.699 (0.513–0.950)	0.028
Mostly male nurses (>75%)	0.555 (0.342–0.821)	0.002	0.628 (0.331–1.082)	0.088	0.523 (0.254–0.906)	0.030
Stature (quartiles)		0.887		0.966		0.869
1	Reference		Reference		Reference	
2	0.817 (0.628–1.036)	0.100	0.727 (0.497–1.030)	0.066	0.960 (0.658–1.349)	0.744
3	1.101 (0.869–1.364)	0.442	1.191 (0.875–1.578)	0.260	1.013 (0.735–1.382)	0.976
4	1.088 (0.834–1.369)	0.522	1.174 (0.842–1.597)	0.370	1.007 (0.675–1.446)	0.946
Team with only registered nurses	0.990 (0.814–1.177)	0.904	1.176 (0.899–1.508)	0.246	0.809 (0.591–1.074)	0.130
Nursing team psychological characteristics						
Neuroticism^a^		0.075		0.432		0.110
1	Reference		Reference		Reference	
2	1.233 (0.989–1.529)	0.060	1.124 (0.850–1.482)	0.440	1.396 (1.004 ‐ 1.900)	0.046
3	1.238 (0.929–1.160)	0.144	1.175 (0.780–1.696)	0.480	1.307 (.824 ‐ 1.938)	0.278
Extraversion^a^		0.015		0.001		0.661
1	Reference		Reference		Reference	
2	1.136 (0.903–1.405)	0.304	1.467 (1.034–2.028)	0.032	0.890 (0.644–1.206)	0.420
3	1.666 (1.210–2.270)	<0.001	2.470 (1.564–3.582)	<0.001	1.000 (0.627–1.498)	0.906
Openness^a^		0.535		0.526		0.810
1	Reference		Reference		Reference	
2	1.147 (0.911–1.568)	0.276	1.272 (0.918–1.736)	0.144	1.028 (0.739–1.423)	0.942
3	1.035 (0.766–1.372)	0.854	1.015 (0.672–1.461)	0.974	1.086 (0.703–1.579)	0.756
Conscientiousness^a^		0.145		0.130		0.368
1	Reference		Reference		Reference	
2	1.249 (0.974–1.568)	0.082	1.409 (0.997–1.943)	0.052	1.159 (0.820–1.571)	0.436
3	1.184 (0.874–1.580)	0.272	1.271 (0.838–1.873)	0.278	1.226 (0.797–1.782)	0.398
Agreeableness^a^		0.776		0.731		0.773
1	Reference		Reference		Reference	
2	1.005 (0.822–1.234)	0.974	1.077 (.810–1.387)	0.662	.931 (.687 ‐ 1.239)	0.606
3	0.947 (0.713–1.236)	0.644	0.863 (0.574–1.246)	0.428	1.016 (.660 ‐ 1.484)	0.986
General feeling of safety^a^		0.226		0.099		0.790
1	Reference		Reference		Reference	
2	1.101 (0.877–1.365)	0.416	1.206 (0.849–1.642)	0.250	0.981 (0.725–1.331)	0.834
3	1.216 (0.925–1.552)	0.162	1.462 (0.975–2.079)	0.078	0.969 (0.640–1.398)	0.768
Shift characteristics						
Day shift	Reference		Reference		Reference	
Evening shift	0.916 (0.766–1.104)	0.306	0.903 (0.718–1.134)	0.340	0.951 (0.727–1.215)	0.640
Night shift	0.290 (0.216–0.381)	<0.001	0.221 (0.142–0.318)	<0.001	0.423 (0.285–0.607)	<0.001
Patient characteristics						
Male	1.161 (0.979–1.358)	0.084	1.225 (0.972–1.521)	0.088	1.053 (0.842–1.299)	0.686
Age^b^	0.893 (0.866–0.920)	<0.001	0.902 (0.866–0.938)	<0.001	0.884 (0.847–0.923)	<0.001
Primary diagnosis						
Psychotic disorder	Reference		reference		Reference	
Bipolar disorder	1.666 (1.370–1.974)	<0.001	1.655 (1.287–2.096)	<0.001	1.636 (0.237 ‐ 2.105)	<0.001
Other diagnosis	1.008 (0.740–0.1341)	0.984	0.413 (0.211–0.698)	0.004	1.712 (1.134 ‐ 2.351)	0.010
Comorbidity						
Substance abuse	0.694 (0.591–0.798)	<0.001	0.715 (0.570–0.881)	<0.001	0.686 (0.538–0.850)	<0.001
Personality disorder	1.499 (1.160–1.891)	<0.001	1.096 (.739–1.529)	0.652	1.899 (1.365–2.517)	<0.001
Intellectual impairment	2.204 (1.792–2.670)	<0.001	2.911 (2.232–3.668)	<0.001	1.248 (0.847–1.734)	0.274
Involuntary admission	4.838 (3.313–7.114)	<0.001	4.210 (2.257–7.469)	<0.001	5.519 (3.281–9.355)	<0.001

Abbreviations: CrI, credible interval; OR, odds ratio.

^a^ Cutoffs at centile 17 and 83

^b^ Effect size of patient's age is reported in age differences (steps) of 5 years.

#### 3.3.1 Nursing team and shift variables

We found that during shifts with teams composed of >75% males, there were fewer incidents of patients' aggression incidents than in shifts with teams composed of females only, OR 0.56 (95% CrI 0.34–0.82). Higher team scores on personality trait extraversion were associated with more aggressive behavior of patients, OR 1.67 (95% CrI 1.21–2.27). Higher team scores on neuroticism showed a nonsignificant trend toward more aggressive behavior, OR 1.23 (95% CrI 0.90–1.53). We observed less aggressive behavior in the night shift compared with the day shift.

#### 3.3.2 Patient variables

Patient characteristics showed relatively strong associations with aggressive behavior of patients. Higher age was associated with less aggressive behavior, OR 0.893 (95% CrI 0.866–0.920) per each 5 year. Those with involuntary admissions were more likely to exhibit aggressive behavior, OR 4.838 (95% CrI 3.313–7.114). The same holds for those with bipolar disorder, comorbid personality disorder, and comorbid intellectual impairment. Comorbid substance abuse was associated with lower probability of showing aggressive behavior.

#### 3.3.3 Verbal and physical aggression

We performed additional analyses distinguishing between verbal and physically aggressive behavior of patients. These analyses showed comparable results to those mentioned above, with a few notable exceptions, namely the associations with extraversion, neuroticism, and general feelings of safety. The association between high team scores on extraversion and more aggressive behavior was primarily due to verbal aggression, OR 2.47 (95% CrI 1.56–3.58). The association between higher team scores (second tertile) on neuroticism and more aggressive behavior was somewhat stronger for physical aggression, OR 1.40 (95% CrI 1.00–1.90). A high team score on feelings of safety of nurses was associated, although not statistically significant, with more verbal aggression, OR 1.46 (95% CrI 0.98–2.08).

## 4 DISCUSSION

We investigated the influence of nursing team, shift, and patient characteristics on the incidence of patients' aggressive behavior in a closed psychiatric ward. Nursing teams with >75% males were associated with less aggressive behavior of patients. Aggressive behavior was least likely during the night shift. High team scores on extraversion were strongly associated with verbal aggression, not with physical aggression. In contrast, a high team score on neuroticism was associated (although not significantly with all categories) with physical aggression, but not with verbal aggression. Furthermore, high team scores on feelings of safety tended to be associated with verbal aggression. We found several patient characteristics (young age, diagnoses other than psychotic disorder, psychiatric comorbidity, and involuntary admission) to be associated with aggressive behavior.

Our finding that aggressive behavior occurs least during night shifts is supported by previous findings (Salzmann‐Erikson & Yifter, 2019). This seems obvious, because patients sleep at night and (potentially) provoking events, such as medication administration, concentrate during the daytime or evenings. We found that young patients and patients who are involuntary admitted have higher odds to show aggressive behavior. This is in line with findings of several systematic reviews (Iozzino et al., 2015; Salzmann‐Erikson & Yifter, 2019; Weltens et al., 2021). Salzmann‐Erikson and Yifter (2019) found evidence in their review for an association of several diagnostic categories with aggressive behavior, such as psychotic disorders, bipolar disorder and personality disorders. Equivocalness in findings of diagnostic categories suggests that these findings are highly sample‐dependent.

We found an association between all female nurses in a team and increased odds of aggressive behavior. Because of the equivocalness of this characteristic in other studies, we are cautious in assuming a causal relationship (Odes et al., 2021). We found associations between higher nursing teams' mean of personality trait extraversion and more verbal aggression and, although less strong, higher nursing team's mean of personality trait neuroticism and more physical aggression. Extravert individuals are characterized by enthusiasm and they can be perceived as dominant in groups of people (McCrae et al., 2005). This may indicate that extravert staff members can be a trigger for patients' aggression. Another possible explanation is that teams with high levels of extraversion actively seek interaction with patients and therefore encounter more verbal aggression but de‐escalate this before exacerbation into physical violence. Neurotic persons are characterized by emotional instability and are sensitive to stress (McCrae et al., 2005). A possible explanation for the association with physical aggression could be a tendency of teams with high levels of neuroticism to be anxious to intervene early in the development of aggression and therefore may encounter more physical aggression. There is little evidence on the association between staff personality trait and patients' aggressive behavior. Bilgin (2009) measured nurses' interpersonal styles with the Interpersonal Style Inventory and reported associations between nurses with less sociable and less tolerant interpersonal styles and physical assault by patients and relatives. Sociable individuals have a preference for working together and they interact with others, and tolerant individuals are generally able to handle stress and provocation more calmly (Bilgin, 2009). This seems in line with our finding that high team levels of neuroticism are associated with more physical aggression. Başoğul et al. (2019) used the sociotropy‐autonomy scale to measure the personality traits of nurses. They found an association between sociotropic personality characteristics and verbal aggression. Sociotropic individuals have good empathy skills and interest in helping others during interpersonal interaction and may be comparable to the agreeableness personality trait, which we found to be not associated with aggressive behavior. However, extrapolation of the personality traits we measured to other models of psychological characteristics is highly speculative. Therefore, we need to be cautious in comparing our findings with results found with other models. Lastly, we found a nonsignificant trend toward a higher team scores of feelings of safety and more verbal aggressive behavior. A possible explanation for this finding, apart from chance, is that teams that generally feel safer tend to seek interaction with patients and therefore encounter more verbal aggression. Future research could evaluate the effects of feelings of safety of staff members on the incidence of aggressive behavior.

Our study has several limitations. We decided to exclude data on patient's current clinical state because of poor data quality. Therefore, we were unable to account for the influence of severity of the disorder on the risk of aggressive behavior. Furthermore, nurses were aware of the fact that we performed a study about aggressive behavior. We cannot rule out that this influenced their behavior or their reporting of aggressive behavior, although we used regular daily nursing reports as a primary data source. We analyzed nursing characteristics at the team level. The cross‐classified data structure limited the possibility to analyze the effect at the level of individual nurses, due to nonconvergence of the statistical model when adding another level. This prevented us from analyzing the influence of individual characteristics of nurses. Due to the complexity of the statistical model, we were not able to analyse interaction variables between patient and team characteristics. This study was conducted in the Netherlands, which possibly limits the generalizability of our findings to other parts of the world. Lastly, this was a monocentric study, which could also limit the generalizability of our findings.

## 5 IMPLICATIONS FOR NURSING PRACTICE

The reported associations may raise nurses' awareness about factors that may increase the probability of aggressive behavior in patients. Our findings suggest that nursing teams with extrovert personalities are more at risk to encounter patient verbal aggression than teams with more introvert nurses are. This might imply that an interaction strategy with low expressed emotions diminishes the risk of verbal aggression. The association between a neurotic personality structure and physical aggression is a new finding (based on a nonsignificant *p* value) and requires replication. Anxious or controlling behavior of nurses might not protect against aggression, perhaps because nurses who feel safe reach out to patients earlier in the development of aggressive behavior. These findings could serve as a starting point for further qualitative (e.g., phenomenological analysis or participative observation of patient–staff interaction) and quantitative research on nurses' personality traits in relation to the patient outcomes. We deem it inappropriate to use current findings for selecting staff members. However, it generates possibly clinically relevant hypotheses concerning the influence of personality traits on aggressive behavior. For instance, improving the insight of nurses in their own personality traits could serve as a starting point for training and development of nurses' interactional skills. Ultimately, this may lead to the development of preventive interventions.

## CONFLICTS OF INTEREST

The authors declare no conflicts of interest.

## ETHICS STATEMENT

Medical Ethics Review Board of the Academic Medical Center in Amsterdam, the Netherlands (reference number A1 ‐ 12 17 0320).

## Supporting information

Supporting information.

Supporting information.

## Data Availability

The data that support the findings of this study are available on request from the corresponding author. The data are not publicly available due to privacy or ethical restrictions.
