# Supplementary material for: Association between characteristics of nursing teams and patients' aggressive behavior in closed psychiatric wards
Source: Perspect Psychiatr Care. 2022 May 3;58(4):2592–600. doi: 10.1111/ppc.13099 (PMC9790403; doi:10.1111/ppc.13099)
Supplement: Supplementary file 1 — Supporting information. [file PPC-58-2592-s001.docx]

**Online supplement 1 - List of variables**

|  | Level | Source | Measurement |
| --- | --- | --- | --- |
| **Nursing staff variables †** |  |  |  |
| Gender | Dichotomous | CRF | Male; female |
| Age | Continuous | CRF | Age on first shift in study period in years |
| Body Mass Index | Continuous | CRF | Weight (in kg) divided by height (in cm) squared |
| Physical stature | Ordinal | CRF | Very small; small; average; large; very large |
| Registered nurse | Dichotomous | CRF | Yes; no |
| Bachelor of nursing | Dichotomous | CRF | Yes; no |
| Full-time work status | Dichotomous | CRF | Yes; no |
| Employment at current ward | Continuous | CRF | Cumulative employment in years |
| Experience in mental health care | Continuous | CRF | Cumulative experience in years |
| Personality traits (NEO-FFI-3) |  |  |  |
| Neuroticism | Continuous | LimeSurvey | Sum of the twelve questions from NEO-FFI-3 that addressed neuroticism |
| Extraversion | Continuous | LimeSurvey | Sum of the twelve questions from NEO-FFI-3 that addressed extraversion |
| Openness | Continuous | LimeSurvey | Sum of the twelve questions from NEO-FFI-3 that addressed openness |
| Conscientiousness | Continuous | LimeSurvey | Sum of the twelve questions from NEO-FFI-3 that addressed conscientiousness |
| Agreeableness | Continuous | LimeSurvey | Sum of the twelve questions from NEO-FFI-3 that addressed agreeableness |
| General feeling of safety during work | Continuous | LimeSurvey | Sum of the four questions that addressed feeling of safety |
| **Shift characteristics** |  |  |  |
| Weekend | Dichotomous | EHR | Yes; no |
| Shift | Nominal | EHR | Day (7:30AM - 4:00PM), evening (2:30PM - 11:00PM), night (10:45PM - 7:45AM) |
| Registered nurses | Continuous | Schedule | Number of registered nurses present at the ward |
| Student nurses | Continuous | Schedule | Number of student nurses present at the ward |
| Patients | Continuous | EHR | Number of patients present at the ward |
| Patient-staff ratio | Continuous | EHR | Number of patients for each nurse present in a shift |
| **Patient characteristics** |  |  |  |
| Gender | Dichotomous | EHR | Male; female |
| Age | Continuous | EHR | Age at admission in years |
| Total length of admission | Continuous | EHR | Duration of admission in days |
| Involuntary admission | Dichotomous | EHR | Yes; no |
| Diagnosis (according to DSM-IV-TR) | Nominal | EHR | Psychotic disorder; bipolar disorder; other disorder than psychotic or bipolar disorder |
| Psychiatric co-morbidity |  |  |  |
| Substance abuse | Dichotomous | EHR | Yes; no |
| Personality disorder | Dichotomous | EHR | Yes; no |
| Intellectual impairment | Dichotomous | EHR | Yes; no |
| Aggressive behaviour prior to admission | Dichotomous | EHR | Yes; no |
| First admission in mental health care | Dichotomous | EHR | Yes; no |
| Global Assessment of Functioning (GAF) | Continuous | EHR | Most recent score on the GAF-scale |
| Health of Nation Outcome Scale (HoNOS) | Continuous | EHR | Most recent score on the HoNOS |
| **Outcome measures (SOAS-R)** |  |  |  |
| Provocation | Nominal | EHR | No understandable provocation; provoked by other patient(s); help by ADL; patient being denied something; staff requiring patient to take medication; other provocations |
| Means used by patient | Nominal | EHR | Verbal aggression; ordinary objects (chair, glass(ware); other objects); parts of the body (hands, feet, teeth, other); dangerous methods or objects (knife, strangulation, other) |
| Target of aggression | Nominal | EHR | Nothing/nobody; object(s); other patient(s); patient self; staff member(s); other persons |
| Consequences for victim(s) | Nominal | EHR | No; objects damaged, replacement not necessary; objects damaged, replacement necessary; persons felt threatened; persons in pain <10 minutes; persons in pain >10 minutes; visible injuries; need for treatment; need for treatment by a physician; other |
| Measures to stop aggression | Nominal | EHR | None; talk to patient; calmly brought away; oral medication; parenteral medication; held with force (physical restraint); seclusion; mechanical restraint; other measures |

† We analysed nursing staff factors aggregated at shift team level. To obtain better contrasts in the explanatory variables, we avoided using dichotomies, and used at least three categories for each variable. The nurses’ gender was analysed in four categories, namely teams with only female nurses, mixed teams with more female nurses (>50%), mixed teams with more male nurses (50-75%) and teams with mostly male nurses (>75%). Teams with only female nurses were the reference category. The variable nurses’ stature was analysed in four about equally sized groups (quartiles) of the mean team stature, using the quartile with the lowest mean stature as reference category. The six nursing team personality characteristics were divided into three categories with cut-offs at the 17^th^ and 83 centiles of the distribution (1/6^th^ 2/3^rd^, and 1/6^th^) using the lowest categories as the reference categories.
