# Supplementary material for: Association between characteristics of nursing teams and patients' aggressive behavior in closed psychiatric wards
Source: Perspect Psychiatr Care. 2022 May 3;58(4):2592–600. doi: 10.1111/ppc.13099 (PMC9790403; doi:10.1111/ppc.13099)
Supplement: Supplementary file 2 — Supporting information. [file PPC-58-2592-s002.docx]

**Online supplement 2 - Stata code for cross-classified multilevel regression analysis**

This file contains the main preparatory commands and the regression commands used in STATA version 15 to run the runmlwin command in MLwiN version 3.02 to obtain the results of this paper

--------------------------------------------------------------------------------------------------------

name: <unnamed>

log: c:\temp\aggression_29preds_20200513.log

log type: text

opened on: 13 May 2020, 13:21:29

* Create four macros containing the predictors at the level of the patient, the time of day, and the team

. *// 1. PATIENTBASIC

. global patientbasic age male dx2 dx3 sub_abuse person_dis int_imp involuntary_current // 8 vars (7 determinants)

* where dx2 indicates bipolar disorder and dx3 other diagnosis than psychotic or bipolar disorders (psychotic disorders being the reference category); sub_abuse denotes substance abuse as comorbidity (yes/no), person_dis denotes if a personality disorder had been diagnosed as comorbidity (yes/no), int_imp denotes intellectual impairment as comorbidity, and involuntary_current denotes if the patient's current admission has been involuntarily

. *// 3. SHIFTBASIC

. global shiftbasic shiftdum2 shiftdum3 // 2 vars (1 determinant)

* where shiftdum2 indicates evening shift and shiftdum3 the night shift

. *// 5. TEAMBASIC

. *egen mean_male_4cats = cut(mean_male), at(0, 0.01, 0.50, 1.00, 1.10)

. tab mean_male mean_male_4cats if pickone_team==1, m

. *egen teamstature = cut(mean_stature), group(4)

. tab teamstature if pickone_team==1

teamstature | Freq. Percent Cum.

------------+-----------------------------------

0 | 266 20.48 20.48

1 | 265 20.40 40.88

2 | 462 35.57 76.44

3 | 306 23.56 100.00

------------+-----------------------------------

Total | 1,299 100.00

. *tab teamstature, g(teamstatureB)

. *gen teamregist = cond(mean_reg_nurse<1,0,1)

. tab teamregist if pickone_team==1

teamregist | Freq. Percent Cum.

------------+-----------------------------------

0 | 387 29.79 29.79

1 | 912 70.21 100.00

------------+-----------------------------------

Total | 1,299 100.00

. global teambasic teamstatureB2 teamstatureB3 teamstatureB4 teammalepctC2 teammalepctC3 teammalepctC4 teamregist // 7 vars (3 determinants)

. *// 6. TEAMEXTENDED

. *drop teamsafe*

. centile mean_safety_sum if pickone_team==1, c(17, 83)

-- Binom. Interp. --

Variable | Obs Percentile Centile [95% Conf. Interval]

-------------+-------------------------------------------------------------

mean_safet~m | 1,287 17 14.75 14.66667 15

| 83 17 17 17

. *egen teamsafety = cut(mean_safety_sum), at(0, 14.75, 17.00, 25)

. tab teamsafety if pickone_team==1, mis

teamsafety | Freq. Percent Cum.

------------+-----------------------------------

0 | 212 16.32 16.32

14.75 | 806 62.05 78.37

17 | 269 20.71 99.08

. | 12 0.92 100.00

------------+-----------------------------------

Total | 1,299 100.00

. *tab teamsafety, g(teamsafeC)

. *// ASSESS THE DISTRIBUTIONS

. *tw hist mean_neuroticism_sum, percent yla(,angle(h))

. *tw hist mean_extraversion_sum, percent yla(,angle(h))

. *tw hist mean_openness_sum, percent yla(,angle(h))

. *tw hist mean_conscientiousness_sum, percent yla(,angle(h))

. *tw hist mean_agreeableness_sum, percent yla(,angle(h))

. for each var of varlist mean_neuroticism_sum mean_extraversion_sum mean_openness_sum mean_conscientiousness_sum mean_agreeableness_sum {

2. centile `var' if pickone_team==1, c(17 83)

3. }

-- Binom. Interp. --

Variable | Obs Percentile Centile [95% Conf. Interval]

-------------+-------------------------------------------------------------

mean_neuro~m | 1,286 17 24.66667 24.33971 25

| 83 30.33333 30 30.66667

-- Binom. Interp. --

Variable | Obs Percentile Centile [95% Conf. Interval]

-------------+-------------------------------------------------------------

mean_extra~m | 1,286 17 40.5 40 41

| 83 48.33333 48 48.5

-- Binom. Interp. --

Variable | Obs Percentile Centile [95% Conf. Interval]

-------------+-------------------------------------------------------------

mean_openn~m | 1,286 17 40 40 40.33333

| 83 46 45.66667 46

-- Binom. Interp. --

Variable | Obs Percentile Centile [95% Conf. Interval]

-------------+-------------------------------------------------------------

mean_consc~m | 1,286 17 42.75 42.5 43

| 83 48 48 48.33333

-- Binom. Interp. --

Variable | Obs Percentile Centile [95% Conf. Interval]

-------------+-------------------------------------------------------------

mean_agree~m | 1,286 17 41.31583 41 41.5

| 83 48 47.66667 48

. *egen teamneurot= cut(mean_neuroticism_sum), at(0, 24.67, 30.33, 60)

. *tab teamneurot, g(teamneurotC)

. tab teamneurot if pickone_t==1, m

teamneurot | Freq. Percent Cum.

------------+-----------------------------------

0 | 228 17.55 17.55

24.67 | 823 63.36 80.91

30.33 | 235 18.09 99.00

. | 13 1.00 100.00

------------+-----------------------------------

Total | 1,299 100.00

.

. *egen teamxtravert= cut(mean_extraversion_sum), at(0, 40.5, 48.33, 60)

. *tab teamxtravert, g(teamxtravertC)

. tab teamxtravert if pickone_t==1, m

teamxtraver |

t | Freq. Percent Cum.

------------+-----------------------------------

0 | 217 16.71 16.71

40.5 | 850 65.43 82.14

48.33 | 219 16.86 99.00

. | 13 1.00 100.00

------------+-----------------------------------

Total | 1,299 100.00

.

. *egen teamopen= cut(mean_openness_sum), at(0, 40, 46, 60)

. *tab teamopen, g(teamopenC)

. tab teamopen if pickone_t==1, m

teamopen | Freq. Percent Cum.

------------+-----------------------------------

0 | 187 14.40 14.40

40 | 880 67.74 82.14

46 | 219 16.86 99.00

. | 13 1.00 100.00

------------+-----------------------------------

Total | 1,299 100.00

. *egen teamconsci= cut(mean_conscientiousness_sum), at(0, 42.75, 48, 60)

. *tab teamconsci, g(teamconsciC)

. tab teamconsci if pickone_t==1, m

teamconsci | Freq. Percent Cum.

------------+-----------------------------------

0 | 216 16.63 16.63

42.75 | 790 60.82 77.44

48 | 280 21.56 99.00

. | 13 1.00 100.00

------------+-----------------------------------

Total | 1,299 100.00

. *egen teamagreeb= cut(mean_agreeableness_sum), at(0, 41.32, 48, 60)

. *tab teamagreeb, g(teamagreebC)

. tab teamagreeb if pickone_t==1, m

teamagreeb | Freq. Percent Cum.

------------+-----------------------------------

0 | 218 16.78 16.78

41.32 | 844 64.97 81.76

48 | 224 17.24 99.00

. | 13 1.00 100.00

------------+-----------------------------------

Total | 1,299 100.00

. global MLwiN_path "C:\Program Files\MLwiN v3.02\mlwin.exe"

. global patientbasic age male dx2 dx3 sub_abuse person_dis int_imp involuntary_current // 8 vars (7 determinants)

. global shiftbasic shiftdum2 shiftdum3 // 2 vars (1 determinant)

. global teambasic teamstatureB2 teamstatureB3 teamstatureB4 teammalepctC2 teammalepctC3 teammalepctC4 teamregist // 7 vars (3 determinants)

. global teamextended teamneurotC2 teamneurotC3 teamxtravertC2 teamxtravertC3 teamopenC2 teamopenC3 teamconsciC2 teamconsciC3 teamagreebC2 teamagreebC3 teamsafeC2 teamsafeC3 // 12 vars (6 determinants)

* SORTING THE DATA FILE

. sort team patid

. *// PQL2 MODEL TO OBTAIN STARTING VALUES FOR THE MCMC MODEL, where PQL indicates 'penalised quasi-likelihood'

. qui runmlwin agres_event cons $patientbasic $shiftbasic $teambasic $teamextended, level2(team: cons) level1(patid) discrete(dist(binomial) link(logit) denom(cons) pql2) or maxit(500) nopause

. *// MCMC MODEL, NOTE THE CC OPTION ("CROSS CLASSIFIED")

. runmlwin agres_event cons $patientbasic $shiftbasic $teambasic $teamextended, level2(team: cons, residuals(u2_1)) level1(patid) discrete(dist(binomial) link(logit) denom(cons) pql2) mcmc(cc burnin(2000) chain(20000) thinning(20)) initsprevious or nopause

MLwiN 3.2 multilevel model Number of obs = 18610

Binomial logit response model

Estimation algorithm: MCMC

-----------------------------------------------------------

| No. of Observations per Group

Level Variable | Groups Minimum Average Maximum

----------------+------------------------------------------

team | 1286 5 14.5 426

-----------------------------------------------------------

Burnin = 2000

Chain = 20000

Thinning = 20

Run time (seconds) = 713

Deviance (dbar) = 6088.10

Deviance (thetabar) = 6019.57

Effective no. of pars (pd) = 68.53

Bayesian DIC = 6156.62

. log close

name: <unnamed>

log: c:\temp\aggression_29preds_20200513.log

log type: text

closed on: 13 May 2020, 13:34:18
